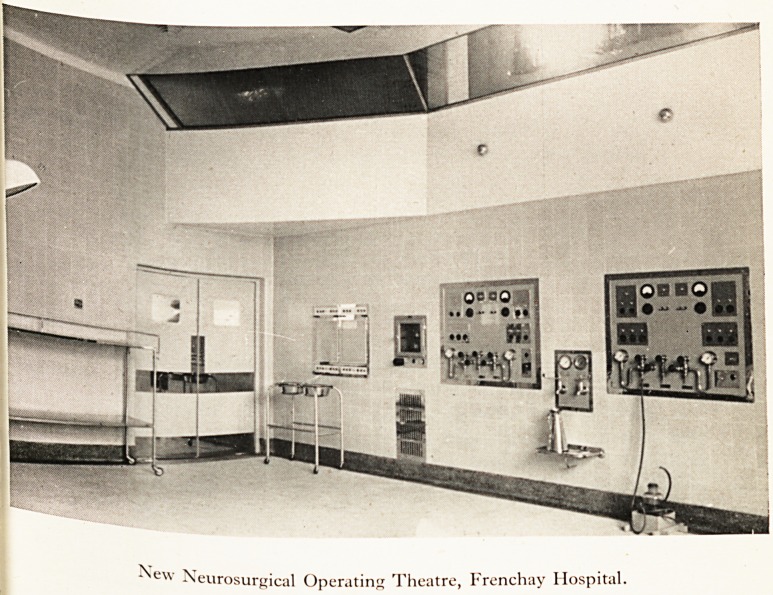# Neurosurgical Unit, Frenchay

**Published:** 1954

**Authors:** 


### NEUROSURGICAL UNIT, FRENCHAY

On 14th October, new theatres and extensions for the department of ne logical surgery at Frenchay Hospital, Bristol, were opened by Professo Geoffrey Jefferson, F.R.S., in the presence of about 500 guests. The Mayor of Bristol, Ald. K. A. Brown, presided. Introductory remarks were by Dr. T. Howard Butler, Chairman of the Management Committee, and b Lord Mayor.

Sir Geoffrey Jefferson remarked that brain surgery was no longer a subje top-line news in the daily press; he thought the emphasis of public interes shifted to the thoracic surgeons, with their cardiac surgery. He welcome new expansion of the facilities for the neurosurgical service of the Region referred to the new premises as a Prospero's dream, but with substance in He described them as probably the finest neurosurgical theatres in existen

Sir Geoffrey noted with pleasure that Professor Norman Dott from Edinb was present, and Miss Beck and Mr. Northfield from London. Mr. Lang from Cardiff was also among the guests at this function. After formally dec the extensions open, and after a benediction by the hospital chaplain Greenup, the symbolic opening of the theatres was performed by the cuttin ribbon with surgical scissors by Sir Geoffrey Jefferson. Mr. G. L. Alex proposed the vote of thanks to Sir Geoffrey Jefferson. The principal then led a tour of inspection of the new buildings, and later tea was ser one of the newly-converted neurosurgical wards.

Among others present were the Lady Mayoress and Lady Jefferson Vice-Chancellor of the University of Bristol, Sir Philip Morris, and Lady M Mrs. Norman Dott; the Sheriff's lady, Mrs. Alan Wills; and represent of the University, the Ministry of Health, the Ministry of Works, an Regional Hospital Board.

The project was one approved on the appointed day and as such was fin directly by the Ministry of Health. The work was carried out under age the Ministry of Works for the most part by local contractors. The layou sists of twin operating theatres and adequate ancillary accommodation. theatres are air-conditioned and other steps have been taken to minimize contamination when operations are in progress. Substantial improveme four wards allocated for neurosurgery have been included in the schem a corridor connecting the wards and theatres has been enclosed. The th will soon be working [*Plate IX*].

PLATE IX

New Neurosurgical Operating Theatre, Frenchay Hospital.

## Figures and Tables

**Figure f1:**